# Recent Progress on Carbon-Dots-Based Probes for Microbial Labeling and Versatile Analysis Applications

**DOI:** 10.3390/bios16030137

**Published:** 2026-02-26

**Authors:** Ying Liu, Ping Yu, Jinhua Li, Yang Liu, Ming Ma, Sihua Qian, Yuhui Wang, Yunwei Wei

**Affiliations:** 1 College of Biological and Environmental Sciences, Zhejiang Wanli University, Ningbo 315100, China; liuying123@nimte.ac.cn; 2 Laboratory of Advanced Theranostic Materials and Technology, Ningbo Institute of Materials Technology & Engineering, Chinese Academy of Sciences, Ningbo 315201, China; yuping@nimte.ac.cn (P.Y.); qiansihua@nimte.ac.cn (S.Q.); 3 Ningbo Customs Technology Center, Ningbo 315048, China; 13736012141@163.com (J.L.); 15867572519@163.com (M.M.); 4 Department of Pancreatic and Gastrointestinal Surgery Division, Ningbo No. 2 Hospital, Ningbo 315010, China; lyang712@icloud.com

**Keywords:** carbon dots, microbial labeling, recognition mechanism, fluorescent imaging, detection

## Abstract

Microbial imbalance and the spread of pathogenic microorganisms pose severe threats to human health and ecological security. Traditional microbial detection methods suffer from several drawbacks such as long detection time, low sensitivity, and insufficient specificity. As an emerging fluorescent probe, carbon dots (CDs) offer an innovative direction for microbial labeling and detection due to their ultra-small particle size, unique optical properties, excellent biocompatibility, and facile surface modifiability. Herein, this article reviews the research progress of CDs on microbial labeling and detection. The content covers a brief introduction of CDs and explores the main recognition strategies including non-covalent interactions and biomolecule-mediated targeted binding. It also elaborates on the application status of multi-modal sensing technologies for microbial detection, such as CDs-based fluorescent sensing, electrochemical sensing, and surface-enhanced Raman scattering (SERS) sensing. Additionally, the challenges faced in current research, such as achieving simultaneous detection of multiple pathogens and in vivo dynamic tracking, are analyzed, and the development prospects of CDs in fields like clinical diagnosis and public health monitoring are prospected. This review aims to provide comprehensive references for further research and application of CDs in the field of microbial detection.

## 1. Introduction

As important components of the human body, microbial imbalance and the spread of pathogenic microorganisms have become major hidden dangers threatening human health and ecological security [1,2]. Data from the World Health Organization (WHO) shows that approximately 700,000 people die from antibiotic-resistant bacterial infections worldwide each year. Among them, “superbugs” such as carbapenem-resistant *Enterobacteriaceae* (CRE) have been classified as the highest-level emergency threat [3,4]. Foodborne pathogenic microorganisms like *Escherichia coli* O157:H7 and *Salmonella* cause about 420,000 deaths globally each year [5], while the pandemic of Severe Acute Respiratory Syndrome Coronavirus 2 (SARS-CoV-2) has further highlighted the catastrophic risks of cross-species transmission of respiratory pathogenic microorganisms [6]. The essence of these hazards lies in the adaptive evolutionary ability of microorganisms at the genomic level. For example, *Helicobacter pylori* colonize the gastric mucosa by secreting urease [7], and influenza viruses evade vaccine protection through hemagglutinin protein mutation [8]. This microscopic survival strategy makes the development of highly sensitive and specific microbial detection technologies a key link in blocking the hazard chain.

Culture methods and biochemical identification are the most classic microbial detection methods [9]. They rely on the growth characteristics of microorganisms in specific culture media and the analysis of metabolites. For instance, the detection of *Salmonella* in food requires a culture cycle of 48–72 h, and it is unable to detect viable but non-culturable microorganisms. Nucleic acid amplification technology has brought detection to the molecular level. Quantitative Real-Time PCR (qPCR) can detect SARS-CoV-2 with a sensitivity of single-copy nucleic acid, but it requires professional laboratories and faces problems such as difficult primer design and susceptibility to interference from sample inhibitors. Immune detection technology realizes rapid detection through the specific binding of antigens and antibodies. The Enzyme-Linked Immunosorbent Assay (ELISA) method has a high sensitivity of ng/mL level, but antibody preparation has a long cycle, high cost, and risks of cross-reaction. Therefore, there is an urgent need to improve existing rapid microbial detection methods. Benefiting from their unique optical properties [10], fluorescent materials play an increasingly important role in microbial sensing and detection. To date, organic dyes [11], quantum dots (QDs) [12], upconversion nanoparticles (UCNPs) [13], and gold nanoclusters (AuNCs) [14] have been gradually adopted for the specific imaging and detection of microbial infections. However, these probes involve some native drawbacks, like poor water solubility, photobleaching, metal toxicity, and low quantum yield [15,16].

Carbon dots (CDs) are a new type of carbon-based fluorescent nanomaterials. Their common subcategories include graphene quantum dots (GQDs), carbon quantum dots (CQDs), carbon nanodots (CNDs), and carbonized polymer dots (CPDs). Due to their many fascinating properties (such as easy synthesis, tunable fluorescence emission, low cost, and good physical and chemical stability), they have attracted extensive attention in the fields of chemical/biological sensing, imaging, catalysis, nanomedicine, and optoelectronic devices [17,18]. Compared with traditional fluorescent probes, such as organic dyes and QDs, CDs possess significant superiorities: facile synthesis and surface modification, excellent water solubility, and strong resistance to photobleaching. In particular, good biocompatibility and high tolerance to photobleaching make CDs competitive candidates in the construction of fluorescent probes [19,20]. At present, research on CDs in the field of microbial labeling and detection has shown great application value, but the relevant achievements have not been summarized. Despite recent reviews about CDs and their microbial detection and imaging applications having been reported [21,22,23], they rarely mention the classification of recognition mechanisms, and multiple-signal sensing technologies. Therefore, it is of great academic and practical significance to review the application of CDs in microbial detection and to integrate the cutting-edge progress of their recognition strategy and multiple-signal sensing technologies.

**Figure 1:**
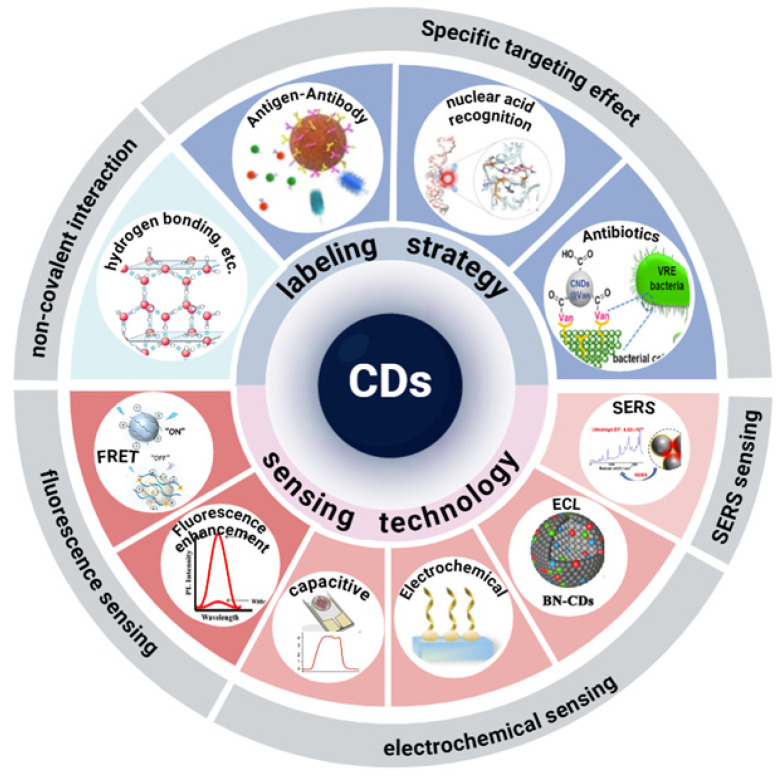
An overview on the binding strategies between fluorescent CDs and microorganisms, as well as CDs-based diverse methods for the detection of microorganisms.

Focusing on CDs, this article conducts a review around the theme of microbial detection: first, it summarizes the synthesis methods of CDs to lay a material foundation for subsequent analysis; then, it focuses on the specific application research of CDs in the labeling, detection, and imaging of microorganisms such as bacteria and viruses, and deeply discusses the material design ideas, interaction mechanisms, and typical application scenarios of CDs in this scenario; finally, combined with existing research, it comprehensively analyzes the core research value of CDs in the field of microbial detection, the current technical challenges, and prospects for future development directions, in order to provide references for subsequent research in this field (Figure 1).

## 2. Synthesis and Brief Introduction of CDs

Carbon dots (CDs), also known as carbon nanodots, are a class of emerging fluorescent nanomaterials. The synthesis of CDs is highly flexible and versatile, with a wide range of precursor materials and reaction routes available to tailor their properties. Common synthesis methods can be broadly divided into two categories: top-down and bottom-up approaches [24,25]. Top-down approaches involve the fragmentation of bulk carbon materials into nanoscale dots, which typically include laser ablation [26,27] arc discharge and chemical oxidation [28,29]. For instance, laser ablation utilizes high-energy laser pulses to ablate carbonaceous materials such as graphite, carbon nanotubes, or activated carbon in liquid media, generating CDs with relatively uniform size distribution [24]. Chemical oxidation method utilizes strong oxidants (e.g., concentrated nitric acid, concentrated sulfuric acid) to etch and cleave carbon precursors, yielding carbon dots with abundant oxygen-containing functional groups on their surfaces [30]. Bottom-up approaches, by contrast, build CDs from small-molecule precursors through polymerization and carbonization processes, including hydrothermal/solvothermal synthesis, microwave-assisted synthesis, and pyrolysis. Hydrothermal synthesis [31,32,33], one of the most widely used methods, conducts the reaction of precursors like citric acid, urea, or glucose in a high-temperature and high-pressure aqueous environment, enabling the formation of CDs with good crystallinity and stable fluorescence [34]. Microwave-assisted synthesis offers the advantages of rapid heating, short reaction time, and high efficiency, making it suitable for large-scale preparation [25,35]. The choice of precursor, reaction temperature, time, and solvent in these synthesis strategies can significantly affect the structural (e.g., sp^2^/sp^3^ hybridization ratio, particle size) and functional (e.g., surface functional groups, optical properties) characteristics of CDs.

As typical quasi-zero-dimensional carbon nanomaterials with a diameter of less than 10 nm, carbon dots (CDs) encompass four main subtypes, namely graphene quantum dots (GQDs), carbon quantum dots (CQDs), carbon nanodots (CNDs) and carbonized polymer dots (CPDs). The core distinctions among these subtypes lie in their carbon skeleton structures, the doping status of heteroatoms, and the species and distribution of surface functional groups [36,37,38,39]. Their unique optical properties make them stand out among traditional fluorescent materials: (1) They exhibit excitation wavelength-dependent fluorescence emission, and their emission spectra can be modulated through precursor engineering, enabling multi-color labeling and ratiometric detection [40,41,42,43]. (2) Their anti-photobleaching ability is 2–3 orders of magnitude higher than that of conventional organic dyes, allowing them to withstand continuous excitation for over 120 min [41,44,45]. (3) Notably, compared to traditional probes such as QDs, CDs exhibit significantly lower cytotoxicity and excellent biocompatibility [46], which is a crucial advantage for biological applications. (4) Carbon dots can act as electron donors or acceptors, promoting the separation and transfer of photogenerated charges [47,48,49]. All these unique optoelectronic properties make CDs promising candidates in chemo/biosensing, imaging, therapy, optoelectronic devices, catalysis, and so on [50,51,52,53,54,55,56,57].

## 3. Recognition Mechanism Between CDs and Microorganisms

**Figure 2:**
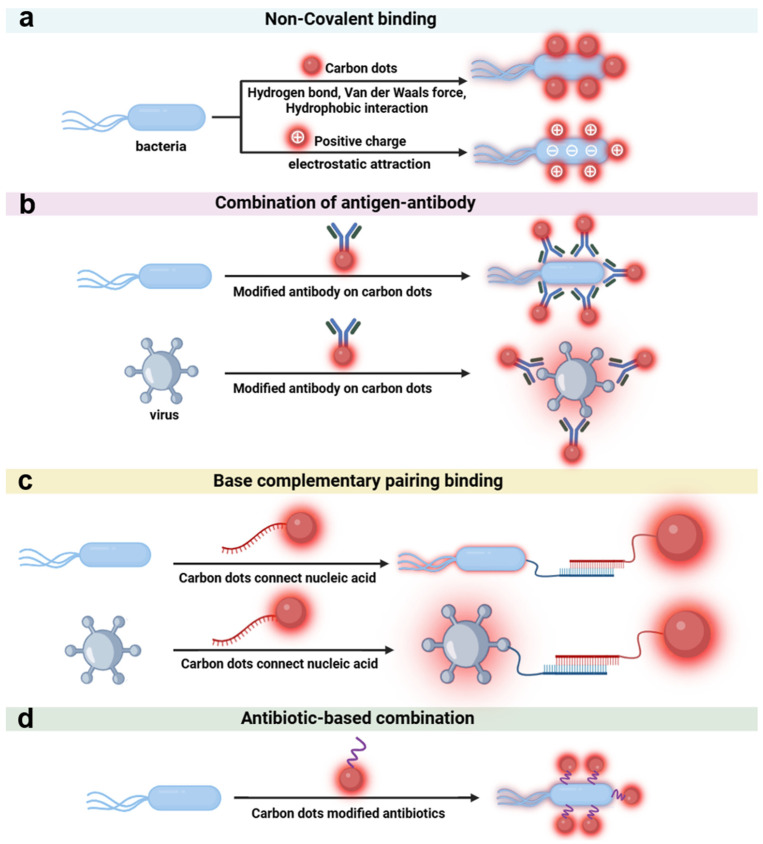
Common combination principles between CDs and microorganism. (**a**) Non-covalent interaction-based binding; (**b**) antigen–antibody-based binding; (**c**) base complementary pairing binding; (**d**) antibiotic-based recognition and binding.

As a nanomaterial with excellent optical properties and surface modifiability, CDs exhibit unique advantages in the field of microbial recognition [58,59]. Their recognition strategies for microorganisms are based on the physical and chemical properties of the material itself and the specific interaction with biomolecules, mainly including the following three mechanisms: (1) Utilizing natural or modified surface functional groups such as hydroxyl, amino, and carboxyl groups to achieve preliminary recognition or broad-spectrum detection of microorganisms through non-covalent interactions such as hydrogen bonding, electrostatic attraction, hydrophobic interaction, and van der Waals forces [60]. (2) Regulating surface charge to achieve selective recognition, especially distinguishing between live and dead cells, by utilizing the charge interaction with microbial cell membranes [61,62]. (3) Achieving precise recognition through the modification of biomolecules such as antibodies and nucleic acid probes [63,64]. The above labeling strategy is illustrated in Figure 2 and discussed below.

### 3.1. Non-Covalent Interaction Recognition Based on Surface Functional Groups

The core of non-covalent binding lies in the hydroxyl, amino, carboxyl, and other functional groups on the surface of CDs, which selectively bind to components such as lipids and proteins in microbial membranes and cell walls through hydrogen bonding, hydrophobic interaction, etc. No additional modification is required, and the operation is simple with low cost, making it suitable for rapid screening [65,66].

The composition of surface functional groups of CDs is closely related to their precursors and synthesis methods, and the type and distribution of functional groups directly determine the type and strength of non-covalent interactions [67]. For example, CDs synthesized by the hydrothermal method using citric acid and urea as raw materials are rich in hydroxyl and amino groups on the surface, which easily bind to microorganisms through hydrogen bonding and electrostatic interaction [68]; CDs synthesized using ascorbic acid and ethylenediamine as raw materials are mainly composed of amino and carboxyl groups, which tend to interact with polar molecules through electrostatic attraction [69]; CDs prepared using plant extracts such as apple juice [70] and papaya juice [71] as raw materials usually have multiple functional groups such as hydroxyl, carboxyl, and amide groups on the surface due to the complex composition of plant components, which can synergistically recognize microorganisms through multiple non-covalent forces.

When CDs use bacterial cells as precursors, their surface functional groups can retain the biological characteristics of the original bacteria, and enhance the recognition ability for similar microorganisms through the synergistic effect of multiple non-covalent forces [72]. For example, *Escherichia coli*-derived CDs (CDs-WT) use *Escherichia coli* cells as raw materials, and the surface retains the carboxyl, amino, and hydroxyl groups of the cell wall of the original bacteria. The synergistic effect of carboxyl electrostatic attraction and amino hydrogen bonding enhances the recognition of similar microorganisms, and at the same time, it can penetrate the cell membrane and exhibit multi-color fluorescence under different excitation lights [73]. Curcumin-based CDs (CDP) use curcumin as a precursor and are synthesized by the hydrothermal method with the assistance of Polyethyleneimine (PEI). The surface of CDP has phenolic hydroxyl, amide, and amino groups at the same time. These functional groups achieve broad-spectrum recognition of Gram-negative and Gram-positive bacteria through differential non-covalent interactions. The labeling efficiency of CDP for *Escherichia coli* and *Staphylococcus aureus* reaches more than 90% [74]. In addition to hydrophilic functional groups, the introduction of hydrophobic functional groups can enhance the non-covalent binding between CDs and cell membranes through hydrophobic interaction, which is especially suitable for the recognition of microbial membrane structures [75]. The hydrophobic part of amphiphilic CDs (M-CDs) binds to the lipid bilayer of the microbial cell membrane through hydrophobic interaction, while the hydrophilic part ensures the dispersibility of CDs in aqueous solution, enabling rapid bacterial morphology detection within 30 s [76].

Based on the non-covalent binding mechanism of surface functional groups, the preliminary recognition of microorganisms by CDs is realized. Its characteristics of strong universality, low cost, and simple operation make it an ideal choice for rapid microbial screening, especially suitable for on-site detection in resource-constrained areas. The non-covalent interactions mediated by different functional groups are complementary, further expanding the recognition range and meeting the detection needs of various microorganisms such as bacteria, viruses, and fungi. However, the universality of non-covalent interactions limits the detection specificity, and charge regulation strategies need to be introduced to distinguish between live and dead bacteria.

### 3.2. Electrostatic Interaction Recognition Based on Surface Charge

**Figure 3:**
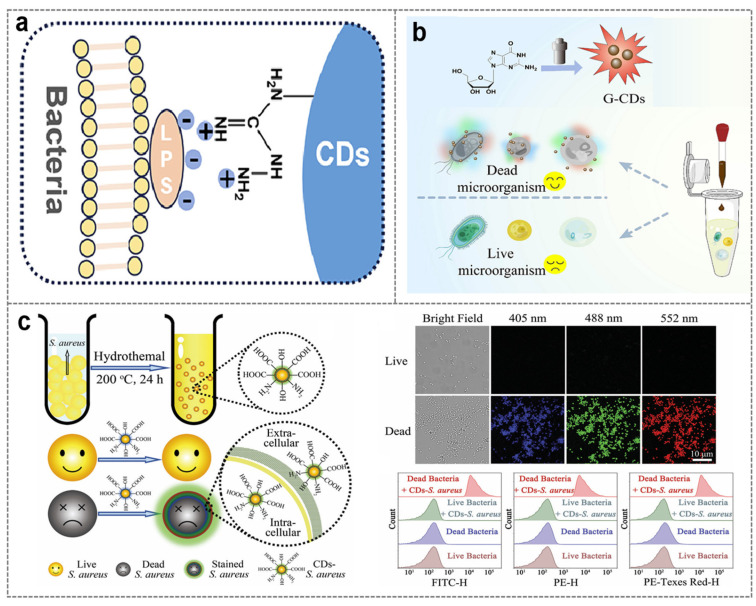
Selective staining of bacteria based on electrostatic interaction. (**a**) Surface charge interaction [78]; (**b**) synthesis of guanosine-based CDs and its application in dead microbial imaging [85]; (**c**) the schematic diagram of the synthesis route of CDs-S and the confocal fluorescence image and flow cytometry analysis of dead bacteria selective staining and the CDs-S staining of live and dead *Staphylococcus aureus* [86].

The surface charge of CDs realizes the selective recognition of microorganisms through charge interaction with the microbial cell membrane [77,78]. Its advantage is that it can quickly distinguish the state of microorganisms by using the difference in microbial membrane potential, with rapid response and no need for complex labeling, making it suitable for scenarios such as microbial viability assessment and antibiotic screening [79,80]. The cell membranes of most bacteria and fungi are negatively charged due to the enrichment of phospholipids and lipopolysaccharides [81], and the capsid proteins or envelopes of virus particles are also mostly negatively charged [82]. The surface charge of CDs usually achieves recognition through the following two ways: first, positively charged CDs combine with negatively charged microbial cell membranes through electrostatic attraction to achieve broad-spectrum recognition; second, negatively charged CDs use the electrostatic repulsion with the cell membrane of live bacteria and the penetration difference after the membrane damage of dead bacteria to distinguish between live and dead states (Figure 3a).

Positively charged CDs can combine with negatively charged microbial cell membranes through electrostatic attraction. For example, cysteamine-derived nitrogen–sulfur co-doped CDs are rich in amino groups on the surface, which can undergo electrostatic attraction with the cell membranes of *Escherichia coli* and *Staphylococcus aureus*, quickly adsorb on the bacterial surface and gradually penetrate. Fluorescent labeling can be achieved within 15 min, with a labeling efficiency of more than 95%, and the penetration into Gram-positive bacteria with loose peptidoglycan layers is stronger [83]. Due to the electrostatic repulsion with the cell membrane of live bacteria, negatively charged CDs cannot penetrate the intact cell membrane; when bacteria die, the membrane integrity is lost, the negative charge density decreases, and CDs can enter the cell and combine with intracellular components to emit bright fluorescence, forming a recognition mode of “bright for dead bacteria, dark for live bacteria”. For example, bacterial extracellular polysaccharide-based CDs are rich in carboxyl and hydroxyl groups on the surface, and have no fluorescent signal for live *Lactobacillus* spp., but show strong green fluorescence for heat-killed *Lactobacillus* spp. This property enables real-time tracking of the ratio of live/dead bacteria during food spoilage [84].

Similarly, guanosine-derived CDs (G-CDs) have negatively charged groups such as carboxyl groups on the surface, with a Zeta potential of −14.5 mV, which can selectively stain dead microorganisms. The cell membrane of live microorganisms is intact and negatively charged, which generates strong electrostatic repulsion with G-CDs to prevent CDs from entering; while the cell membrane of dead microorganisms is damaged, the negative charge barrier disappears, and G-CDs can penetrate and combine with intracellular components, showing multi-color fluorescence under different excitation wavelengths such as 405 nm, 488 nm, and 552 nm (Figure 3b) [85]. A more typical example is that *Staphylococcus aureus*-derived CDs (CDs-*S. aureus*) have extremely high surface negative charge, with a zeta potential of −42.4 mV. The electrostatic repulsion between CDs-*S. aureus* and live bacteria is extremely strong, making it impossible to enter live bacteria; however, due to the damaged cell membrane of dead bacteria, CDs-*S. aureus* can freely penetrate and combine with intracellular DNA, emitting blue, green, and red fluorescence under excitation at 405 nm, 488 nm, and 552 nm respectively, realizing multi-channel live/dead distinction (Figure 3c) [86].

Based on the electrostatic interaction mechanism, by regulating the surface charge of CDs, broad-spectrum capture of microorganisms or specific distinction between live and dead states can be achieved. However, it lacks selectivity and it is hard to target specific pathogens. Thus, more specific targeting molecules need to be introduced to CDs achieve precise recognition.

### 3.3. Specific Recognition Based on Biomolecular Modification

To enhance specificity, researchers have further introduced biomolecular modification strategies, endowing CDs with “molecular-level” precise targeting capabilities for target microorganisms through highly specific biological recognition such as antigen–antibody reactions and nucleic acid hybridization [87,88]. This strategy not only significantly improves the specificity and sensitivity of detection but also provides reliable technical support for clinical diagnosis and epidemic prevention and control. The following will sort out the specific targeted recognition mechanism of CDs based on biomolecular modification and their typical applications.

#### 3.3.1. Antigen–Antibody-Mediated Recognition

The antigen–antibody reaction is one of the most widely used methods in molecular recognition. The principle is that the antibody modified on the surface of CDs can form a spatially complementary structure with the epitope of the antigen on the microbial surface, thereby specifically binding and forming an immune complex. The affinity of this binding is much higher than that of non-specific interactions; based on this, CDs also have been adopted for antibody labeling and microorganism detection [89].

**Figure 4:**
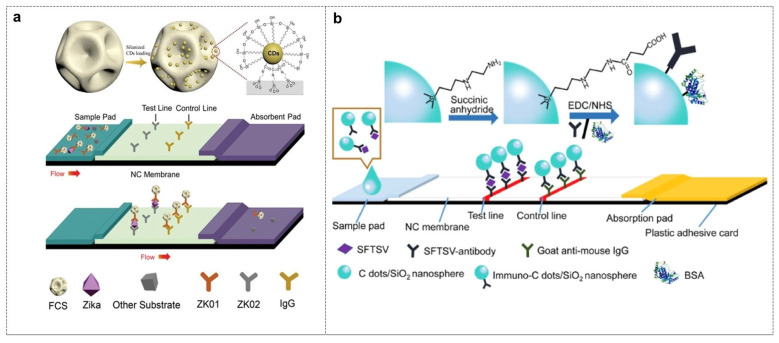
Microbial detection of CDs based on antigen–antibody recognition mechanisms. (**a**) Schematic of iFSC-based lateral flow immunoassay for detecting Zika NS1 protein [90]; (**b**) schematic of ultra-sensitive detection of hemorrhagic fever with thrombocytopenia syndrome virus using immunofluorescent carbon dots/SiO_2_ nanospheres [91].

Zika Virus (Zika Virus) NS1-specific antibodies were covalently immobilized on the surface of dendritic silica–carbon dot composites (iFCS) to prepare fluorescent probes. The NS1 antigen in the sample first undergoes primary specific binding with the antibody on iFCS, then migrates to the detection line under the action of chromatography, and is secondarily recognized by the capture antibody, forming a “capture antibody-NS1-iFCS” sandwich structure. The two specific antigen–antibody recognitions ensure that the target molecule is accurately “sandwiched” in the middle. The fluorescence intensity of the enriched CDs is proportional to the NS1 concentration, with a visual detection limit as low as 10 pg/mL, and the sensitivity is 100 times that of traditional gold-labeled test strips (Figure 4a) [90]. Using the same lateral chromatography structure, only the antibody pair is replaced: the surface of carbon dot/SiO_2_ nanospheres (iCSNs) is modified with SFTSV monoclonal antibody (SmAb), which undergoes primary specific binding with the viral nucleoprotein (NP); it is captured by SmAb again at the detection line, forming a “SmAb-NP-iCSNs” sandwich, with a detection limit as low as 10 pg/mL (Figure 4b) [91]. To further improve the sensitivity, another layer of antibody–gold nanoparticles (Au NPs) is introduced outside the sandwich structure. The NLPs antigen first binds to the CD-labeled antibody, then is captured by another type of antibody modified with non-spherical gold nanoparticles, forming a multi-layer sandwich of “CDs–antibody–NLPs–antibody–Au NPs”. The antigen–antibody reaction is still the only driving force for capture and immobilization, and the local surface plasmon resonance effect of Au NPs acts as a secondary signal amplifier to achieve highly sensitive detection of Norovirus (NoV)-like particles [92].

In general, by coupling with antibodies, CDs can accurately recognize antigens on the surface of bacteria or viruses, achieving precise targeting of target microorganisms and high sensitivity [93]. At the same time, through integrating quick detection technology like immunochromatographic test strips, they can achieve on-site instant detection. Therefore, when CDs are modified with antibodies, they can serve as probes for highly specific detection of microorganisms, and would provide great potential in clinical diagnosis and public health monitoring [94,95].

#### 3.3.2. Specific Labeling Based on Antibiotic Modification

In recent years, the strategy of modifying carbon dots (CDs) with antibiotics for specific bacterial recognition has garnered increasing attention. As recognition elements, antibiotics offer advantages of low cost, excellent stability, and good targeting specificity, making them particularly suitable for the detection of bacteria. Recently, antibiotics-modified CDs have been designed and successfully applied for microorganism labeling and detection.

**Figure 5:**
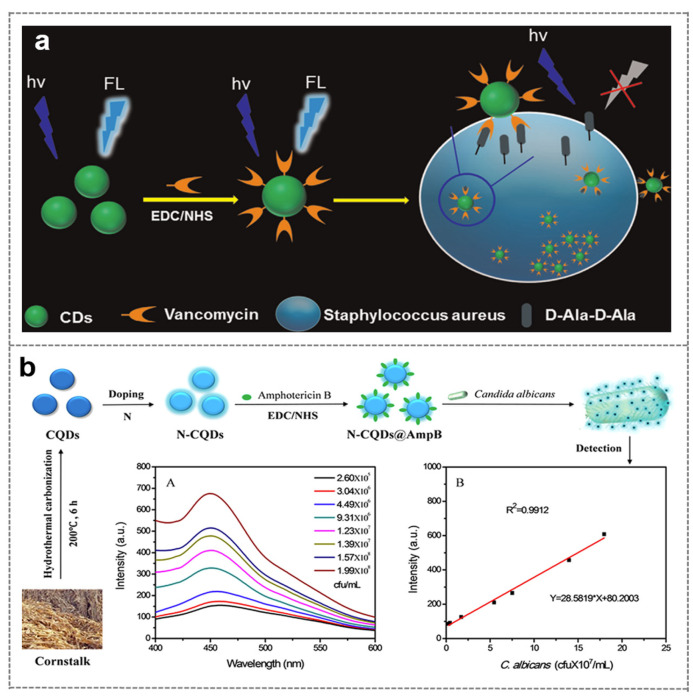
Microbial detection based on antibiotic-mediated recognition. (**a**) The design principle of Van-modified CDs for the detection of *S. aureus* [97]. (**b**) Preparation of N-CQDs@AmpB and its schematic diagram for the detection of *Candida albicansz*.

Vancomycin (Van), a glycopeptide antibiotic, can specifically bind to the D-Ala-D-Ala peptide moieties on the cell wall of Gram-positive bacteria, thereby enabling efficient recognition of target bacteria [96]. Zhong et al. first covalently modified vancomycin onto the surface of CDs to construct a fluorescent probe (CD@Van) for the detection of *Staphylococcus aureus*. Through the specific binding between vancomycin and the bacterial cell wall, the probe induces the aggregation of CDs on the bacterial surface, resulting in quenched fluorescence signals and thus achieving quantitative detection of the target bacterium (Figure 5a). This method exhibits a linear detection range of 3.18 × 10^5^–1.59 × 10^8^ CFU/mL for *Staphylococcus aureus* with a limit of detection (LOD) of 9.40 × 10^4^ CFU/mL. It also demonstrates excellent selectivity with no significant response to Gram-negative bacteria. Furthermore, the method shows good recovery rates (>90%) in complex food matrices such as orange juice, indicating its potential application in real sample analysis [97]. Tabaraki et al. further developed nitrogen and chlorine co-doped carbon dots (N,Cl-CDs) and modified their surface with vancomycin to construct a highly sensitive fluorescent probe for *Staphylococcus aureus* (*S. aureus*). The probe displays a favorable linear response in the concentration range of 10^2^–10^7^ CFU/mL with an ultra-low LOD of 10 CFU/mL. Compared to unmodified CDs, N,Cl-CDs@Van exhibits significantly enhanced recognition capability for target bacteria and excellent anti-interference performance against various interfering bacteria (e.g., *Escherichia coli*, *Klebsiella pneumoniae*). The study also points out that the introduction of vancomycin not only strengthens the binding affinity between CDs and bacterial cell walls but also further improves labeling efficiency through electrostatic interactions [98]. Furthermore, a novel pH-responsive vancomycin-modified carbon nanodots system (CNDs@Van) can achieve targeted recognition of vancomycin-resistant enterococci (VRE), expanding the application of antibiotic-modified carbon dots in the detection of drug-resistant bacteria [99]. In this system, Van was covalently cross-linked on the surface of carbon nanodots to form a polymer structure, which could target the D-alanyl-D-lactic acid group at the end of peptidoglycan of the VRE cell wall and enhance the specific binding of VRE biofilm.

Amphotericin B (AmpB), a polyene antifungal antibiotic, can specifically bind to ergosterol on the cell membrane of fungi such as *Candida albicans*, thereby enabling efficient recognition of target fungi [100]. Yu et al. synthesized CQDs by hydrothermal carbonization using corn straw as a green carbon source, and obtained N-CQDs with better fluorescence performance by ethylenediamine doping. Then, water-soluble or alcohol-soluble AmpB was modified on the surface of N-CQDs to form N-CQDs@AmpB composite probe. The probe was based on the specific binding of AmpB to ergosterol on the cell membrane of *Candida albicans* to achieve fluorescence signal response. The detection linear range of the water-soluble probe was 2.60 × 10^5^ to 1.99 × 10^9^ CFU / mL, and the detection limit was as low as 1124 CFU/mL. This method has short detection time, strong specificity, and good recovery rate in complex samples such as beef sausage. It overcomes the shortcomings of time-consuming traditional culture methods and the high costs of PCR technology, and provides a new way for rapid and sensitive detection of *Candida albicans* (Figure 5b) [101].

In addition, researchers have also explored physicochemical approaches to enhance the conjugation efficiency between antibiotics and carbon dots (CDs). For instance, Kumar et al. employed a two-step sonochemical method to conjugate ampicillin and chloramphenicol with polyethylene glycol-derived carbon dots without the use of catalysts, fabricating fluorescent nanocomposites with a particle size ranging from 4 to 7 nm. This study demonstrated that compared with free drugs, such nano-antibiotic composites exhibited stronger antibacterial activity against multidrug-resistant (MDR) bacteria, with the bactericidal effect of the chloramphenicol composite being enhanced fourfold. More importantly, benefiting from the excellent fluorescent properties of carbon dots, the composites successfully enabled real-time bioimaging of drug-resistant *Escherichia coli* (*E. coli ATCC*) and *DH5α* strains, allowing clear visualization of the drug internalization process into recipient bacterial cells. This work thus provides a dual-functional nanoplatform for the clinical diagnosis and targeted therapy of drug-resistant bacteria [102].

In another study focusing on the preparation and functionalization of carbon quantum dots (CQDs) using antibiotics, researchers investigated in depth the interaction mechanisms between antibiotics and CDs as well as their applications in biosensing. For example, when antibiotics such as amikacin and colistin were used as precursors or functionalizing agents, the resulting antibiotic-modified CQDs showed enhanced specificity against Gram-negative bacteria including *E. coli*. Amikacin-modified CQDs were synthesized via the hydrothermal carbonization of amikacin and diammonium citrate, which possessed a high nitrogen content due to N-doping from amikacin, thereby enhancing their photoluminescent properties. These CQDs could selectively bind to *E. coli* through electrostatic and hydrogen-bonding interactions with the negatively charged lipopolysaccharides (LPSs) on the bacterial surface, achieving a limit of detection (LOD) of 552 cfu/mL. Similarly, colistin-functionalized CQDs prepared by the solid-state carbonization of ammonium citrate and colistin sulfate utilized the specific binding between colistin and LPS, exhibiting a lower LOD of 460 cfu/mL for *E. coli* detection in complex samples such as human urine and apple juice [103,104,105].

In summary, antibiotic-modified CDs not only enable efficient and specific category discrimination of microorganisms but also can be extended to antibacterial therapy and other fields. They thus demonstrate broad application prospects in food safety, clinical diagnosis, and anti-infective treatment.

## 4. Application of CDs-Based Multiple-Signal Sensing Technologies in Microbial Detection

Apart from common fluorescence character, CDs also exhibit other physical properties like electrochemistry and photoelectric conversion due to the unique electronic properties like electron donors or acceptors. And so, they can serve as multi-channel probes for microbial detection based on fluorescence, electricity, and surface-enhanced Raman scattering (SERS), and their sensing mechanisms and applications are introduced and discussed as below.

### 4.1. Fluorescence Sensing Technology

CDs-based fluorescence technologies for microorganisms are mainly classified into the “On–Off” mechanism and enhanced mechanism. Their principles are designed based on the changes in characteristic parameters such as fluorescence intensity and the emission wavelength of CDs that are induced by the microorganisms. Its core principle is that the recognition event between CDs and microorganisms, such as the aggregation of CDs due to specific binding or the separation of CDs from fluorescence quenchers, triggers changes in the fluorescence properties of CDs, thereby realizing the detection of target microorganisms [106,107]. The outstanding advantages of this technology include high sensitivity, non-invasive detection, and visualization, making it suitable for on-site rapid detection and imaging analysis [108,109].

#### 4.1.1. Fluorescence “On–Off” Mechanism

The fluorescence quenching and recovery mechanism is based on the “On–Off” mode: in the absence of target microorganisms, CDs interact with quenchers, and the fluorescence is quenched; when target microorganisms are present, the recognition event separates CDs from quenchers, and the fluorescence is recovered. The advantage of this mechanism is low background signal, making it suitable for the detection of complex samples. And thus, this mechanism has been widely applied for microbial detection, e.g., HTLV-1 gene detection, and bacterial and viral detection [110].

**Figure 6:**
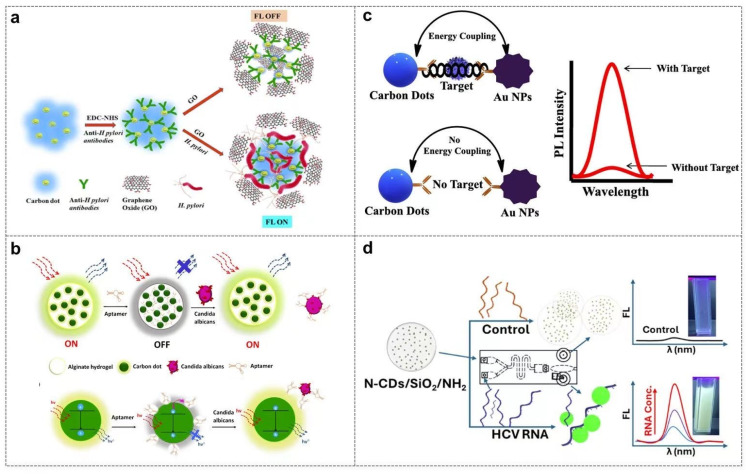
CDs-based fluorescence sensing technologies for microbial detection. (**a**) Schematic illustration of FCD-Ab-GO-based whole-cell *Helicobacter pylori* immunosensor [111]; (**b**) the design principle of alginate-CD-apta-switch nanocomposite for *Candida albicans* identification [113]; (**c**) schematic illustration of non-spherical gold nanoparticle-enhanced carbon dot fluorescence for Norovirus-like particle detection [92]; (**d**) schematic illustration of microfluidic silica-encapsulated CDs for fluorescence enhancement detection of HCV RNA [116].

For example, functionalized CDs (FCDs) were coupled with anti-*Helicobacter pylori* antibodies via the EDC/NHS chemical method to form FCD-Ab probes. In the absence of target bacteria, i.e., *Helicobacter pylori*, FCD-Ab binds to graphene oxide (GO) through π-π stacking, and the strong fluorescence quenching effect of GO turns off the fluorescence of the system. When *Helicobacter pylori* is present, the specific binding force between the antibody and the bacterial surface antigen is much stronger than the interaction with GO, so FCD-Ab detaches from the surface of GO, and the fluorescence is recovered (Figure 6a) [111].

Similarly, in the detection of human papillomavirus type 16 (HPV 16), CDs prepared from sunflower seed shells (SCDs) were modified with polyethyleneimine (PEI) to become positively charged, and bound to negatively charged single-stranded DNA probes through electrostatic interaction. Dabcyl quenched the fluorescence of SCDs via the fluorescence resonance energy transfer mechanism. When HPV 16 DNA is present, it bound to D-DNA through base complementary pairing, causing D-DNA to detach from the surface of SCDs, and the fluorescence of SCDs was recovered. The detection limit of this system was as low as 0.47 nM, making it suitable for trace detection of samples such as urine and cervical swabs [112]. In addition, alginate–carbon dot nanocomposites (Alginate-CD) that were modified with aptamers show high affinity for (1→3)-β-D-glucan on the surface of fungal cell walls and were designed as a probe for the detection of *Candida albicans*. In the absence of *Candida albicans*, the aptamers could lead to the aggregation of CDs through π-π stacking and hydrogen bonds, thus quenching the fluorescence emission of CDs. When *Candida albicans* was present, the aptamers showed a stronger binding force with glucan far away from the surface of CDs, and the fluorescence was recovered. This system had a fast detection speed, a detection limit as low as 40 cells/mL, and no cross-reaction with other fungi (Figure 6b) [113].

#### 4.1.2. Fluorescence Enhancement Mechanism

Fluorescence enhancement mechanisms for microbial detection usually mention the plasmon effect and the aggregation–disaggregation of CDs. These strategies can greatly enhance detection sensitivity [114,115].

For example, in the detection of Norovirus-like particles (NLPs), based on the exciton–plasmon interaction to enhance the fluorescence signal, the synthesized CDs have a fluorescence emission peak at 440 nm, and the absorption peak of non-spherical gold nanoparticles (Au NPs) is at 590 nm. The spectral matching between the two can produce a plasmon enhancement effect. When NLPs are present, CDs and Au NPs were close to each other through antigen–antibody specific binding, and the LSPR property of Au NPs significantly enhanced the fluorescence emission of CDs, with a detection limit as low as 80.3 pg/mL, and the sensitivity was 10 times higher than that of commercial diagnostic kits (Figure 6c) [92]. Similarly, in the detection of hepatitis C virus (HCV) RNA, the aggregation of carbon dots (CDs) is the core fluorescence enhancement mechanism. The positively charged amino-functionalized silica-encapsulated nitrogen-doped carbon dots (N-CDs/SiO_2_/NH_2_) strongly bind to the anionic phosphate backbone of HCV RNA through electrostatic interactions, driving its arrangement and aggregation into a rigid cluster structure. This aggregation triggers the cross-linking enhanced emission (CEE) effect, inhibits the non-radiative decay pathways of CDs (e.g., free rotation and vibrational cascade) and increases the absorption cross section. N-CDs effectively capture excitons, significantly prolong the fluorescence lifetime (τ_1_ = 1.96 ns, τ_2_ = 6.02 ns in the presence of RNA and τ_1_ = 1.49 ns, τ_2_ = 4.30 ns in the absence of RNA), and enhance the fluorescence signal. This strategy can directly detect unamplified HCV RNA in clinical samples, reaching a detection limit of 500 IU/mL on a 3D-printed microfluidic chip and a detection limit of 1000 IU/mL on a 96-well plate. It has high sensitivity (96.47%) and specificity (98.79%) in 141 patient samples (Figure 6d) [116].

The fluorescence quenching and recovery mechanism reduces background interference through the “On–Off” mode, making it suitable for complex samples; the fluorescence enhancement mechanism breaks through the detection limit through signal amplification, making it suitable for trace analysis. Together, they expand the clinical application scenarios of fluorescent sensing in the detection of viruses, bacteria, and fungi, and have irreplaceable advantages especially in on-site rapid imaging and trace screening, laying a methodological foundation for high-throughput and ultra-sensitive detection of multiple types of microorganisms in complex samples.

#### 4.1.3. Nucleic Acid Hybridization-Based Fluorescence Sensing

Nucleic acid-labeled CDs realize microbial nucleic acid detection through the base complementary pairing principle, which is a typical application of nucleic acid-mediated recognition in fluorescence sensing [117,118]. The core principle is that CDs coupled with nucleic acid probes form a double-stranded structure with target microbial nucleic acids, triggering changes in fluorescence properties (e.g., aggregation-induced emission enhancement, fluorescence resonance energy transfer quenching/recovery) to achieve quantitative detection [110,119].

For example, Sheffield et al. [68] reported a fluorometric sensing method for SARS-CoV-2 RNA using N-gene-complementary antisense-oligonucleotide directed molecular aggregation of dual-color carbon dots as probes. In the design, a specific probe was first constructed through the conjugate modification between antisense oligonucleotides (ASOs) and CDs (ASO1 targeting the 421–440 sequence of the viral N gene was coupled with yellow CDs (yCD) to form a Y1 probe; ASO2 targeting the adjacent 443–462 sequence was coupled with blue CDs (bCD) to form a B2 probe). This “adjacent targeting” design ensures hybridization accuracy through sequence position specificity. When SARS-CoV-2 RNA is present in the sample, Y1 and B2 aggregate through base complementary pairing with RNA bridging, triggering the aggregation-induced emission enhancement effect, resulting in significant enhancement of the fluorescence signals of yCD at 540 nm and bCD at 450 nm. The dual-wavelength signal can effectively distinguish interference samples without complementary sequences, reduce false positive risks through cross-validation, and eliminate complex RNA extraction steps, with a detection limit of 81 copies/μL, suitable for early viral infection screening.

In addition, for the gene detection of Human T-cell Leukemia Virus Type 1 (HTLV-1), a fluorescent sensing system based on the “steric hindrance” effect was designed: with two specific probes targeting the 122-base fragment of the tax region of the HTLV-1 genome as the core, one probe was covalently bound to fluorescent CDs. In the absence of target DNA, the single-stranded probe was adsorbed on the surface of Fe_3_O_4_@Au nanoparticles through electrostatic interaction, and the fluorescence of CDs was quenched due to Fluorescence Resonance Energy Transfer (FRET); when HTLV-1 target DNA is present, it forms a double-stranded structure with the two probes through complementary pairing, which cannot be adsorbed on the surface of Fe_3_O_4_@Au due to steric hindrance, and the fluorescence of CDs is restored after being away from the quencher. The linear detection range of this system is 10–320 nM, and the detection limit is less than 10 nM, which can intuitively reflect the content of HTLV-1 genes through fluorescence intensity changes, providing technical support for the early warning of adult T-cell leukemia [120].

### 4.2. Electrochemical Sensing Technology

Electrochemical sensing technology focuses on the charge transport performance of CDs, directly converting microbial recognition events into readable electrical signals, and has the advantages of rapidity, quantification, and real-time monitoring [121]. CDs have excellent conductivity and abundant surface functional sites, enabling rapid electron transfer at the electrode interface [122]. When target microorganisms or their biomarkers interact with CDs through nucleic acid hybridization, antigen–antibody binding, or metabolite interaction, the charge distribution and electron transfer rate at the electrode/solution interface change accordingly, resulting in quantifiable signals such as capacitance, impedance, or electrochemiluminescence (ECL) [123]. This method has a response time of seconds, a wide linear range, a detection limit as low as the single-molecule level, and can be combined with portable electrochemical workstations to achieve in situ dynamic monitoring [124].

#### 4.2.1. Capacitance Change Response

**Figure 7:**
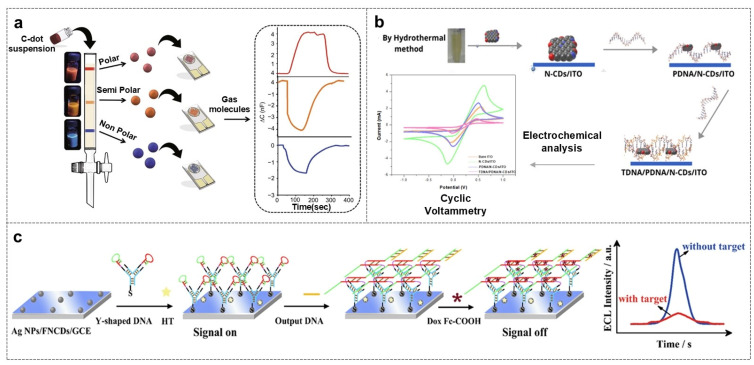
CDs-based electrochemical sensing technologies for detecting microorganisms. (**a**) Schematic illustration of the preparation and response mechanism of a carbon dot-based capacitive gas sensor [126]; (**b**) the present HPV-18 DNA biosensor design based on the immobilization of the probe DNA and hybridization of target DNA onto the N-CD-Modified ITO glass electrode [138]; (**c**) the construction of the ECL sensor substrate based on AgNPs/FNCDs/GCE, and the trigger signal quenching response after the amplification of the target DNA, to achieve the ‘signal on–off’ response diagram [135].

The capacitance change response mechanism is built on the basis of the changes in electrode capacitance before and after the interaction between CDs and microbial metabolites [125]. For example, in an “artificial nose” system, high-polarity, medium-polarity, and low-polarity CDs were sequentially deposited on the surface of interdigital electrodes to construct a polar gradient interface. When *Escherichia coli* grows, the released volatile organic compounds (VOCs) preferentially bound to high-polarity CDs, replacing the water molecules adsorbed on the surface of CDs, resulting in an increase in electrode capacitance, while the non-polar VOCs released by *Staphylococcus aureus* bind to low-polarity CDs, resulting in a decrease in capacitance. By recording the capacitance change combination of the three electrodes, a unique “capacitance fingerprint” could be formed. Combined with a multi-label random forest model, the recognition accuracy of Gram-negative/positive bacteria reached 96%, and the response time was less than 5 min (Figure 7a) [126]. This capacitance change response realized the specific recognition of microbial metabolites by constructing a polar gradient interface, without directly capturing the microorganisms themselves, making it suitable for real-time monitoring of microbial growth status.

#### 4.2.2. Electrochemical Impedance Response

Electrochemical Impedance Spectroscopy (EIS) uses charge transfer resistance (Rct) as a probe, enabling a label-free femtomolar-level quantitative detection of nucleic acid hybridization events [127,128]. For example, in the detection of HPV-18, probe DNA (PDNA) is immobilized on the surface of Indium Tin Oxide (ITO) electrodes modified with nitrogen-doped CDs (N-CDs). The introduction of high conductivity N-CDs made the initial charge transfer resistance of the electrode as low as 18.55 Ω. When PDNA hybridized with target DNA (TDNA) to form a double strand, the negative charge density increased, hindering electron transfer, and also Rct increased to 60.42 Ω. ΔRct had a linear response with TDNA concentration in a range of 0.1 fM–100 pM, and the detection limit was as low as 0.405 fM (Figure 7b) [129]. The electrochemical impedance response directly reflected the binding events of biomolecules through resistance changes, and was especially suitable for trace nucleic acid detection, with important application value in the early diagnosis of diseases such as cervical cancer. However, it has high requirements for the uniformity of electrode modification and is easily affected by the conductivity of the complicated sample matrix.

#### 4.2.3. Electrochemiluminescence (ECL) Response

Electrochemiluminescence (ECL) is a light-emitting process excited through electrochemical reactions, showing remarkable merits of high sensitivity, low background, quick response, ease of operation, and low cost of equipment [130,131]. Owing to the outstanding advantages like unique electrochemical properties, huge specific surface area, abundant defects and chemical groups, CDs are considered as excellent candidates for ECL sensor materials [132]. To date, many CDs with ECL properties have been developed for highly sensitive microbial detection [133].

For instance, boron–nitrogen co-doped CDs (BN-CDs) exhibit significantly higher ECL efficiency compared to non-doped or single-doped CDs, attributed to the synergistic effect of boron (B) and nitrogen (N) doping. Specifically, N doping narrows the band gap of CDs to enhance ECL efficiency, while B doping generates boron radicals (B·) upon electrical excitation. To further amplify the ECL signal and meet the demand for ultra-sensitive biomolecule detection, we constructed a ternary ECL system with S_2_O_8_^2−^ as the coreactant and platinum nanoflowers (Pt NFs) as coreaction accelerators. As a highly efficient electrocatalytic nanomaterial, Pt NFs can accelerate the electron transfer process at the electrode interface—together with B·, they synergistically promote the reduction of S_2_O_8_^2−^ to abundant SO_4_^−^, thereby substantially boosting luminescence intensity. For hepatitis B virus DNA (HBV-DNA) detection, a biosensor constructed by modifying electrodes with BN-CDs and coupling with exonuclease III (Exo III)-induced target DNA amplification strategy achieves a linear ECL intensity response to HBV-DNA concentrations ranging from 100 aM to 1 nM, with an ultra-low detection limit of 18.08 aM [134]; similarly, fluorine–nitrogen co-doped CDs (FNCDs) have been developed as stable and efficient ECL emitters for human immunodeficiency virus DNA (HIV-DNA) detection. The co-doping of fluorine (F) and nitrogen (N) enables FNCDs to form strong hydrogen bonds, which hinder free molecular rotation, reduce non-radiative transitions, and increase the probability of radiative transitions—thus enhancing ECL intensity and stability. In the presence of HIV-DNA, Exo III-assisted multiple recycling amplification converts trace target DNA into abundant output DNA, triggering a horizontal hybridization chain reaction (H-HCR) to form a Y-supported three-dimensional (3D) network DNA nanostructure. This nanostructure immobilizes a large number of doxorubicin-ferrocenecarboxylic acid (Dox-FcCOOH) quenching agents, which significantly reduce the ECL signal of FNCDs. The resulting biosensor exhibits a linear detection range for HIV-DNA of 10 aM to 10 pM, with a detection limit as low as 6.34 aM (Figure 7c) [135].

The ECL sensing strategy integrates the precise regulation of electrochemistry with the high sensitivity of luminescence detection. By optimizing heteroatom co-doping (e.g., B-N, F-N) to enhance ECL emitter performance and introducing signal amplification strategies (e.g., Exo III-induced target recycling, H-HCR), the detection limit can be further minimized—making it highly valuable for clinical early diagnosis and therapeutic effect monitoring. Collectively, the complementary advantages of capacitance, impedance, and ECL sensing modes lay a methodological foundation for the development of multi-parameter, chip-level rapid microbial detection systems.

#### 4.2.4. Photocurrent Response Method

The photocurrent response is a microbial sensing mechanism constructed based on the photocatalytic properties of carbon dots (CDs). Leveraging CDs’ excellent photogenerated charge separation and transfer capabilities, it can rapidly convert microbial recognition events into quantifiable electrical signals. Possessing the advantages of fast response, simple operation, and no need for complex signal labeling, it is suitable for on-site real-time detection scenarios.

The photocurrent response is based on the photocatalytic properties of CDs. When CDs absorb visible light, they generate photo-generated electron-hole pairs, and electron transfer triggers a current change [136]. In the detection of Enterococcus faecalis, a glassy carbon electrode modified with Au@Fe_3_O_4_/CDs generates a large number of photo-generated electron-hole pairs under visible light irradiation, and CDs, as an electron transport channel, significantly improve conductivity. When Enterococcus faecalis is captured on the electrode surface, the redox-active groups such as sulfhydryl and amino groups on its surface are oxidized by photo-generated holes, generating an anodic photocurrent proportional to the number of bacteria. The linear range of this sensor is 1–14 CFU/mL, and the detection limit is as low as 3 CFU/mL. It can directly detect Enterococcus faecalis in fish meat and soil samples without enrichment steps, making it suitable for on-site food safety detection [137].

In summary, through the combination of CDs’ photocatalytic properties and electrode sensing functions, the photocurrent response technology breaks through the dependence of traditional detection on sample pretreatment and complex labeling, and exhibits unique advantages in the rapid quantitative detection of low-concentration microorganisms. In the future, the application scenarios of this technology in the detection of various pathogens can be further expanded by optimizing the structural design of CDs composite electrodes and improving the efficiency of photogenerated charge separation, thereby enhancing detection sensitivity and stability.

### 4.3. Surface-Enhanced Raman Scattering (SERS) Sensing Technology

Surface-enhanced Raman scattering (SERS) sensing technology utilizes the synergistic effect of CDs and the noble metal nanomaterials such as gold and silver. CDs enrich biomolecules on the surface of target microorganisms, and at the same time, the local surface plasmon resonance of gold/silver nanomaterials generates a “hot spot” effect, significantly enhancing the Raman signal of biomolecules on the microbial surface, thereby realizing the detection of microorganisms [138,139]. Its advantages include: the ability to distinguish different microorganisms and even subtypes through the characteristic peaks of Raman spectroscopy; strong anti-interference ability, suitable for complex samples; significant signal amplification effect; and high detection sensitivity [138].

**Figure 8:**
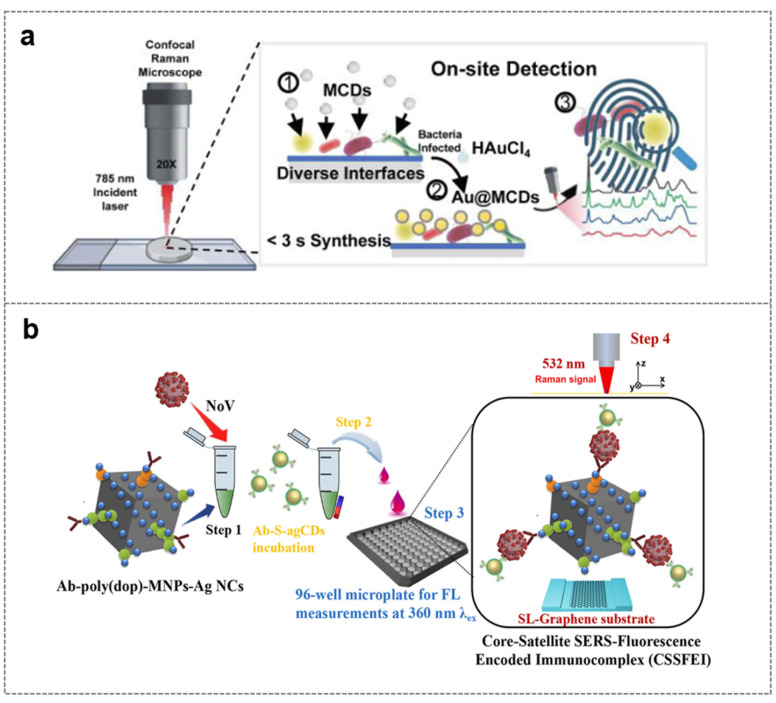
Surface-enhanced Raman scattering (SERS) sensing technology based on carbon dots for microbial detection. (**a**) Schematic illustration of the rapid in situ preparation of Au@MCDs for on-site SERS detection of diverse interfacial pathogens [140]; (**b**) schematic of surface-enhanced Raman scattering detection of Norovirus [141].

In specific applications, gold–carbon dot nanocomposites (Au@MCDs) consist of molybdenum-doped gallic acid-derived carbon dots (MCDs) and gold nanoparticles (Au NPs). MCDs act as both a reducing agent and a stabilizing agent, enabling the in situ reduction of Au^3+^ to form a core–shell (Au core-MCDs shell) structure within 3 s at ambient temperature. The negative charge on the surface of MCDs binds to the positive charge on bacterial surfaces through electrostatic interaction, while π-π stacking facilitates the enrichment of biomolecules on bacterial cell walls. The synergistic effect of the localized surface plasmon resonance (LSPR) of Au NPs and the charge transfer (CT) mechanism of MCDs results in a remarkable Raman signal enhancement factor (EF) of 3.97 × 10^6^ (Figure 8a) [140]. Building on this, the magnetically controlled “core-satellite” dual-probe system further elevates detection sensitivity to the ultra-trace RNA level. For instance, when the sulfur-doped agar-derived carbon dots@polydopamine-functionalized magnetic silver nanocubes (S-agCDs@poly(dop)-MNPs-Ag NCs) system is used for Norovirus (NoV) detection, magnetic silver nanocubes first capture NoV or its virus-like particles (NoV-LPs) to form primary immune complexes. After enrichment via an external magnetic field, these complexes undergo a secondary immune reaction with S-agCDs conjugated to anti-NoV antibodies, leading to the formation of a “core-satellite” immunocomplex (CSSFEI). As the NoV concentration increases, the fluorescence (FL) signal of S-agCDs is synchronously enhanced with the SERS signal: the FL mode achieves a limit of detection of ~80 RNA copies/mL, suitable for rapid preliminary screening; the SERS mode realizes accurate quantification by tracking “hotspot” regions on a single-layer graphene substrate, with an ultra-low LOD of ~10 RNA copies/mL. In the detection of NoV in clinical fecal specimens, the system demonstrates 98% consistency with reverse transcription-polymerase chain reaction (RT-PCR) results (Figure 8b) [141].

The gold–carbon dot composite system realizes the rapid enrichment and signal enhancement of microorganisms through material design, taking into account both detection speed and sensitivity, and is suitable for public health emergency monitoring. However, the accuracy of its “fingerprint recognition” depends on the clear analysis of characteristic peaks, which has high requirements for spectral analysis capabilities; the dual-modal SERS-fluorescence response combines the rapidity of fluorescence detection with the high specificity and quantitative accuracy of SERS, and optimizes the detection process through the “screening first, quantification later” mode.

## 5. Conclusions and Outlook

As an emerging fluorescent nanomaterial, carbon dots (CDs) have shown break-through progress in the fields of microbial detection, imaging, and tracking. This article reviews the application paths of CDs in the labeling of various microorganisms such as bacteria, viruses, and fungi. In summary, relying on core advantages such as ultrasmall size, high photostability, tunable emission, and easy surface modification, CDs have successfully broken through the bottlenecks of traditional detection methods in terms of sensitivity, specificity, timeliness, and biocompatibility. Through the green synthesis strategies of “top-down” and “bottom-up” dual paths, researchers have been able to precisely regulate the carbon core structure, heteroatom doping, and surface functional groups of CDs, realizing multi-color emission from ultraviolet to near-infrared, and meeting the needs of multi-target and multi-channel detection in complex samples.

At the level of the recognition mechanism, this study formed a broad-spectrum-resolution-precision three-level recognition system: (1) Relying on the non-covalent interaction between surface functional groups such as carboxyl, amino, hydroxyl and microbial membrane/cell wall to achieve rapid broad-spectrum capture. (2) Distinguishing live bacteria and dead bacteria by surface charge difference, and provide real-time feedback for antibacterial effect evaluation. (3) Through the modification of biological molecules such as antibodies and nucleic acid probes, the single-copy level accurate detection of single pathogens such as Zika virus, SARS-CoV-2 and Norovirus can be achieved. The system has the characteristics of low background, high signal-to-noise ratio and strong field adaptability, which is obviously superior to traditional culture, PCR, ELISA and other methods. For a more comprehensive understanding, we further summarized CDs-based microorganism detection principles, performance, and application fields, and the results are shown in Table 1.

However, there are still key challenges in CD-based microbial detection before large-scale industrialization and clinical popularization: (1) Simultaneous detection of multiple pathogens: Most existing studies focus on single targets, which are difficult to meet the needs of clinical mixed infections or foodborne epidemic traceability. A library of CDs with different fluorescence lifetimes/wavelengths can be encoded, and high-throughput multi-pathogen screening can be achieved by combining spectral-time unmixing algorithms. (2) In vivo dynamic tracking: Data on the distribution, metabolism, excretion, and long-term toxicity of CDs in animal bodies are still limited. A standardized pharmacokinetic–toxicological evaluation system needs to be constructed, and degradable CDs or externally triggered release systems need to be developed to ensure the safety of clinical transformation. (3) Specific binding: It is of paramount significance to delve into the interaction and binding mechanisms between CDs and microorganisms, encompassing an exploration of the influencing factors such as size, surface properties, and hydrophilicity and hydrophobicity. (4) Application in complex samples: CDs encounter obvious application bottlenecks in complex matrices (blood, feces, food, etc.): proteins and blood cells in blood cause non-specific binding and signal interference; fecal samples have complex impurities and microorganisms that affect specific recognition and are hard to pretreat; fats, proteins and vitamins in food matrices adsorb CDs or inhibit sensing reactions, reducing detection sensitivity and accuracy.

Looking ahead, future research endeavors ought to focus on constructing comprehensive structure–function correlation models, which can effectively address the key challenges mentioned above. These models should be underpinned by artificial intelligence (AI) and deep learning techniques, facilitating high-throughput screening and enabling rational structural optimization—specifically, optimizing the size, surface properties, and hydrophilicity/hydrophobicity of CDs to enhance their specific binding with target microorganisms and reduce non-specific interactions. To solve the bottleneck of complex sample application, pretreatment technologies such as selective centrifugation, magnetic separation, or surface-modified CDs with anti-interference capabilities can be developed to resist the adsorption and interference of proteins, fats, impurities, and other components in blood, feces, and food matrices, thereby improving detection sensitivity and accuracy. For the simultaneous detection of multiple pathogens, the AI-driven structure–function models can assist in encoding a library of CDs with different fluorescence lifetimes/wavelengths, and combining spectral-time unmixing algorithms to achieve high-throughput multi-pathogen screening. To sum up, CDs have gradually moved from “fluorescent bright spots in the laboratory” to “usable, accessible, and affordable” microbial detection tools. Only by implementing cost, throughput, toxicology, and data chains one by one, and targeting solving the existing challenges, can they truly become daily tools for primary disease control, clinical frontlines, and the global public health system.

## Figures and Tables

**Table 1:** A summary on CDs-based probes for microbial labeling and detection.

Probes	Fluorescence Quantum Yield	Methods	Binding Strategy	Targets	Application	Performance (LOD, Specificity, and Detection Time)	Refs.
CDs-WT	15.8%	Fluorescence	Electrostatic action	*Escherichia coli*	In vitro imaging of microorganisms (bacteria, fungi)	/	[73]
CDP	8.607%	Fluorescence	non-covalent interaction	*Escherichia coli*, *Staphylococcus aureus*	Clinical, food bacteria detection	/	[74]
M-CDs	/	Fluorescence	hydrophobic interaction	Gram-negative bacteria: *Escherichia coli*; Gram-positive bacteria: *Staphylococcus aureus*	Rapid detection of bacterial morphology	≤30 s	[76]
Cy@CD	13.2%	Fluorescence	action of static electricity	*Escherichia coli*, *Staphylococcus aureus*	Early diagnosis of bacterial infection	15 min	[83]
G-CDs	14.52%	Fluorescence	action of static electricity	*Escherichia coli*, *Staphylococcus aureus*, *Candida albicans*	Identification of microbial death state	/	[85]
CDs-*S. aureus*	7.0%	Fluorescence	action of static electricity	*Staphylococcus aureus*	Wound infection detection	/	[86]
iFCS	/	Fluorescence	combination of antigen–antibody	Zika Virus	Zika virus infection diagnosis, epidemic monitoring	10 pg/mL	[90]
iCSNs	56.3%	Fluorescence	combination of antigen–antibody	Severe fever with thrombocytopenia syndrome virus	Fever with thrombocytopenia syndrome diagnosis, epidemic monitoring	10 pg/mL	[91]
CDs-AuNP-NLP	48%	Fluorescence	combination of antigen–antibody	Norovirus	Norovirus infection screening, diagnosis	80.3 pg/mL	[92]
yCD-ASO1, bCD-ASO2	/	Fluorescence	Nucleic acid hybridization	SARS-CoV-2	Screening and diagnosis of novel coronavirus infection	81 copies/μL	[68]
CDs-HTLV-1tax	/	Fluorescence	Base complementary pairing binding	Human T-cell leukemia virus type 1	Early diagnosis of HTLV-1 infection and early warning of adult T-cell leukemia	<10 nM	[120]
CD@Van	/	Fluorescence	Antibiotic-based binding	*Staphylococcus aureus*	Food safety detection, clinical diagnosis of *Staphylococcus aureus* infection	9.40 × 10^4^ CFU/mL	[97]
N,Cl-CDs@Van	38.75%	Fluorescence	Antibiotic-based binding	*Staphylococcus aureus*	Food safety detection, clinical diagnosis	10 CFU/mL	[98]
CNDs@Van	/	Fluorescence	Antibiotic-based binding	Vancomycin-resistant enterococcus	Anti-infective treatment of VRE biofilms	/	[99]
N-CQDs@AmpB	37%	Fluorescence	Antibiotic-based binding	*Candida albicans*	Detection of *Candida albicans*	1124 CFU/mL	[101]
CDs-ampicillin	37%	Fluorescence	Antibiotic-based binding	Multidrug-resistant bacteria	Eradication of multidrug-resistant bacteria; bioimaging	MIC = 11 μg/mL (*E. coli ATCC*); 6000 μg/mL (*Dh5α-ampicillin R*)	[102]
CQDs	/	Fluorescence	Antibiotic-based binding	*Escherichia coli* (Gram-negative)	Detection of *E. coli* in PBS and commercial mineral water	460 CFU/mL	[105]
FCD-Ab	/	Fluorescence	Antigen–antibody binding	*Helicobacter pylori*	Clinical diagnosis of *Helicobacter pylori* infection	10 CFU/mL	[111]
SCDs-PEI	23.38%	Fluorescence	Nucleic acid hybridization	Human papillomavirus type 16	Early diagnosis of HPV 16 infection	0.47 nM	[112]
Alginate-CD	4.1%	Fluorescence	Aptamer binding	*Candida albicans*	Clinical diagnosis of *Candida albicans* infection	40 cells/mL	[113]
N-CDs/SiO_2_/NH_2_	/	Fluorescence	Electrostatic interaction	Hepatitis C virus	Clinical diagnosis of HCV infection	500 IU/mL	[116]
Polar gradient CDs	/	Electrochemical	Non-covalent interaction	*Escherichia coli*, *Staphylococcus aureus*	Real-time monitoring of microbial growth	<5 min	[126]
N-CDs/ITO	/	Electrochemical	Nucleic acid hybridization	Human papillomavirus type 18	Early diagnosis of cervical cancer (HPV-18 infection)	0.405 fM	[129]
BN-CDs	/	Electrochemical	Nucleic acid hybridization	Hepatitis B virus	Early diagnosis of HBV infection, therapeutic effect monitoring	18.08 aM	[134]
FNCDs	/	Electrochemical	Nucleic acid hybridization	Human immunodeficiency virus	Early diagnosis of HIV infection	6.34 aM	[135]
Au@MCDs	/	SERS	Electrostatic interaction + π-π stacking	Pathogenic bacteria (general)	On-site SERS detection of diverse interfacial pathogens	10 CFU/mL	[140]
S-agCDs@poly(dop)-MNPs-Ag NCs	/	SERS + Fluorescence	Antigen–antibody binding	Norovirus (NoV)	Clinical detection of norovirus infection	10 RNA copies/mL (SERS); 80 RNA copies/mL (Fluorescence)	[142]
Au@Fe_3_O_4_/CDs	/	Electrochemical	Non-covalent interaction	*Enterococcus faecalis*	Food safety detection	3 CFU/mL	[137]

## Data Availability

All data discussed are derived from published studies cited in the references, which are accessible via standard academic databases.
